# A non-coding role for trypanosome *VSG* transcripts in allelic exclusion

**DOI:** 10.1093/nar/gkaf1011

**Published:** 2025-10-21

**Authors:** Douglas O Escrivani, Sebastian Hutchinson, Michele Tinti, Jane E Wright, Catarina A Marques, Joana R C Faria, Anna Trenaman, David Horn

**Affiliations:** Biological Chemistry & Drug Discovery, School of Life Sciences, University of Dundee, Dow Street, Dundee DD1 5EH, United Kingdom; Biological Chemistry & Drug Discovery, School of Life Sciences, University of Dundee, Dow Street, Dundee DD1 5EH, United Kingdom; Biological Chemistry & Drug Discovery, School of Life Sciences, University of Dundee, Dow Street, Dundee DD1 5EH, United Kingdom; Biological Chemistry & Drug Discovery, School of Life Sciences, University of Dundee, Dow Street, Dundee DD1 5EH, United Kingdom; Biological Chemistry & Drug Discovery, School of Life Sciences, University of Dundee, Dow Street, Dundee DD1 5EH, United Kingdom; Centre for Parasitology, School of Infection and Immunity, University of Glasgow, G12 8TA, United Kingdom; Biological Chemistry & Drug Discovery, School of Life Sciences, University of Dundee, Dow Street, Dundee DD1 5EH, United Kingdom; Biology Department and York Biomedical Research Institute, University of York, YO10 5DD, United Kingdom; Biological Chemistry & Drug Discovery, School of Life Sciences, University of Dundee, Dow Street, Dundee DD1 5EH, United Kingdom; Biological Chemistry & Drug Discovery, School of Life Sciences, University of Dundee, Dow Street, Dundee DD1 5EH, United Kingdom

## Abstract

Bloodstream-form African trypanosomes display antigenic variation. This requires mono-telomeric but switchable expression of a Variant Surface Glycoprotein (VSG) gene in a transcription and splicing compartment that is inter-chromosomally bridged by VSG exclusion factors 1 and 2 (VEX1-2). The dominant gene produces 10 000 times more transcript than excluded *VSGs*. Additional chromatin and RNA-associated factors are required to maintain *VSG* exclusion, but our understanding of the mechanisms involved remains incomplete. Here, we show that the *VSG* transcript impacts allelic competition. We induced either specific translation blockade by recruiting MS2 coat protein to the active *VSG* 5′-untranslated region, or *VSG* transcript depletion using RNA interference. Neither perturbation substantially compromised exclusion of native *VSGs*. In contrast, a *VSG* transgene escaped exclusion specifically when the native transcript was transiently depleted. While both perturbations blocked cytokinesis, DNA replication and mitosis continued when the transcript, which is stabilized by a cyclin-like F-box protein, was translationally blocked. The proportion of nuclei with a second VEX2 focus was significantly increased in cells with a second active *VSG*. We conclude that the *VSG* transcript is a bifunctional coding and non-coding RNA that participates in allelic competition to establish exclusion, a form of RNA-mediated symmetry breaking that also remodels nuclear architecture.

## Graphical Abstract

**Graphical Abstract:**
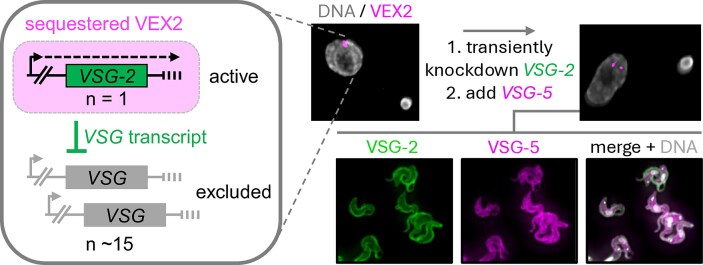

## Introduction

Trypanosomatids are flagellated protozoa and include several vector-transmitted parasites that impact both human and veterinary health. The African trypanosome, *Trypanosoma brucei*, is transmitted by tsetse flies, and causes lethal human and animal diseases. *Trypanosoma brucei* is exclusively extracellular and presents a paradigm for studies on antigenic variation, which underpins evasion of host adaptive immune responses [1, 2]. In preparation for transmission to a mammalian host, *T. brucei* activates Variant Surface Glycoprotein (VSG) expression in the tsetse fly salivary gland, where cells initially express multiple *VSGs* prior to establishing monoallelic expression [3]. The active and many silent bloodstream-form *VSG* expression sites are subsequently stably inherited, with estimates of switching frequency consistently substantially below 1% of cells per generation [4, 5].

The VSG is a super-abundant and essential protein, and each bloodstream-form cell surface is coated with ∼10 million copies. Indeed, the active *VSG* gene accounts for ∼10% of the total cellular messenger RNA (mRNA) and protein. Typically, a single active *VSG* is transcribed by RNA polymerase I in an extranucleolar compartment known as the Expression Site Body, or ESB [6], although two simultaneously active *VSGs* can also share an ESB [7]. Despite the presence of fifteen competent, promoter-associated, telomeric, and mostly polycistronic *VSG* expression sites in the 427 strain used here [8], the active *VSG* produces ∼10 000 times more mRNA than silent *VSGs* [9]; transcription is initiated at all expression site promoters but is attenuated at excluded sites [10]. This extreme form of transcriptional dominance and monogenic expression operates in the context of an (inter-chromosomal) RNA polymerase I transcription and splicing compartment that integrates a telomeric *VSG* and an RNA *trans*-splicing locus [11–13].

Several proteins contribute to maintaining monogenic *VSG* expression. These include positive regulators of *VSG* transcription, ESB1 [14] and SUMOylation [15], and CFB2, a cyclin-like F-box protein that binds and stabilizes *VSG* transcripts [16, 17]. The VSG exclusion (VEX) complex [18, 19] is required to maintain exclusion and forms an inter-chromosomal protein bridge that connects the (VEX2-associated) *VSG* transcription and (VEX1-associated) splicing sub-compartments [12, 13]. Also required to maintain exclusion are the telomere and RNA-binding protein, RAP1 [20, 21], and its interaction with PIP5Pase [22]. In addition, the histone tri-methyltransferase DOT1B is required to rapidly silence inactivated *VSGs* [23, 24], and both the chromatin chaperone CAF-1 [18, 25] and cohesin [26] promote stable inheritance of the active *VSG;* CAF-1 does so by binding the VEX complex [18].

Super-abundant *VSG* mRNA incorporates a highly conserved ‘16-mer’ sequence in its 3′-untranslated region (UTR), and this motif has been implicated in binding RAP1 and antagonizing RAP1-based silencing [20], in binding CFB2 [17], and in promoting *N*^6^-methyladenosine (m^6^A) modification in the poly(A) tail [27]; both m^6^A and CFB2 stabilize the mRNA. Transcription of a second *VSG* driven by T7 phage RNA polymerase induces silencing of the active *VSG* [23], but *VSG* mRNA knockdown fails to induce activation of silent *VSGs* [28]. Specific transcription blockade at the active *VSG* locus does induce activation of silent *VSGs* [29], however; consistent with a maintenance mechanism involving transcription-dependent sequestration of the limiting VEX complex [12], or ESB1 [14], at the *VSG* transcription and splicing compartment.

Despite substantial advances in our understanding of monogenic *VSG* expression control in recent years, our understanding of how the known regulators detailed above establish and maintain *VSG* transcriptional dominance remains incomplete. To further explore allelic competition, and specifically to address the role of the *VSG* transcript, we generated strains in which translation of the active *VSG* could be conditionally and transiently blocked. Using native and transgene *VSG* expression assays, we compared these strains to *VSG* mRNA knockdown strains. Using the transgene assay, we identified a specific exclusion defect associated with transient transcript knockdown, indicating that establishment of *VSG* exclusion is *VSG* transcript dependent.

## Materials and methods

### Trypanosome cell culture

Wild-type, 2T1 [30], and derivative bloodstream-form Lister 427 cells were cultured in HMI-11 medium supplemented with 10% fetal bovine serum (FBS) (not heat inactivated) at 37°C with 5% CO_2_. 2T1 cells were genetically manipulated using cytomix, and a nucleofector II (Lonza) with 0.2 mm cuvettes (Bio-Rad), as previously described [31]. MCP^VSG-2^ and RNAi*^VSG-2^* cells were subcloned and checked for the expected severe growth defect prior to analysis. For the *VSG-5* transgene assay, parental 2T1, MCP^*VSG-2*^, and RNAi*^VSG-2^* cells were induced with tetracycline for 3 h. Cells were then transfected with the *VSG-5* reporter [19]. Cells were washed three times with HMI-11 to remove any residual tetracycline and selected with geneticin (G418) for five to eight days. For tagging VEX2 at the C-terminus, RNAi*^VSG-2^* cells were transfected with HpaI-digested pNAT.VEX2^12myc^ [13]. Antibiotic selection was applied at 10 μg·ml^−1^ blasticidin, 2 μg·ml^−1^ geneticin, 1 μg·ml^−1^ puromycin or phleomycin, and 5 μg·ml^−1^ hygromycin B. Inducible expression systems were activated using tetracycline, which was applied at 1 μg·ml^−1^.

### Plasmid construction

The MCP tandem dimer (tdMCP) sequence was obtained from Addgene plasmid #40649 (phage-ubc-nls-ha-tdMCP-gfp) [32]. The sequence was polymerase chain reaction (PCR)-amplified using the primers MS2CP-LaNLS-HA-HindIII-F (CCCC *AAGCTT* ATG CGAGGACACAAGCGGTCACGTGAA TACCCCTACGACGTGCCCGACTACGCC) and MS2GFPR (GCTA *GGATCC* TTACTTGTACAGCTCGTCCATGC). The SV40-NLS sequence at the N terminus of MCP was replaced with a *T. brucei* La-NLS (underlined in primer and encoding RGHKRSRE [33]) to generate pMCP^GFP^. This fragment was cloned in pRPa^xGFP^ [34] using the HindIII and BamHI sites (italics). A *BLA* selectable marker cassette was PCR-amplified with FWD_BLA_MS2 (CTAGT *GGATCC* TCTAGATGGGTCCCATTG) and with the *VSG-2* start codon within an *Sph*I site (italics) and an MS2 hairpin sequence [35] (underlined) and a portion of the β-tubulin 5′-UTR in the reverse primer REV_MS2_BLA (GAAG *GCATGC* TGTTCTCCAGTTTTGTGTT CTTAAGGCCTGATGGTCCTTAAG TAGATAATTTCGACTATTTTCTTTGATGAAAG). The PCR product was digested with BamHI and SphI (italics) and ligated to a sequence targeting the *VSG-2* gene, digested with BglII and SphI to generate pVSG^MS2^. The MCP^GFP^ and VSG^MS2^ constructs were digested with AscI and XhoI/HindIII, respectively, prior to sequential transfection, to first generate an MCP^GFP^ strain and then to generate the MCP*^VSG-2^* strains. The stem-loop VSG RNAi construct was derived from the pRPa^iSL^ vector [34]. A ∼500 bp fragment of the *VSG-2* gene was PCR-amplified using the primers *VSG-2SL-F* (GATCTCTAGAGGATCCGAGGAGCTAGACGACCAAC) and *VSG-2SL-R* (GATCGGGCCCGGTACCATAGTGACCGCTGCAGAAA).

### *VSG-2* RNA analysis

RNA was isolated from whole cell extracts using the Qiagen RNeasy kit. Reverse transcription of mRNA was performed using M-MLV reverse transcriptase (Promega). First-strand complementary DNA (cDNA) synthesis of *VSG* was primed with a 3′-UTR primer, DH3 (GACTAGTGTTAAATATATCA), while PCR was with DH3 and a ‘spliced leader’ primer, SL22 (GAACAGTTTCTGTACTATATTG). Sanger sequencing was performed using either a spliced leader primer or a *VSG-2*-specific primer. Northern blotting was performed using standard protocols. Two micrograms of total RNA were run on a reducing (1.2% formaldehyde) agarose (1.5%) gel and stained with ethidium bromide. A ∼500 bp *VSG-2* fragment was radiolabelled with ^32^P dCTP (Perkin Elmer) using the large Klenow subunit and random primers for 15 min at 37°C. Probes were hybridized overnight. We used a storage phosphor screen and visualized blots using a Fujifilm FLA-500 image reader.

### Protein blotting

For western blot analysis, 1 × 10^7^ cells were lysed and solubilized in 1 × SDS sample buffer containing 0.1 M DTT at 55°C for 20 min. Proteins were resolved by sodium dodecyl sulfate–polyacrylamide gel electrophoresis (SDS–PAGE) (∼5 × 10^5^ cell equivalents/lane) on NuPAGE bis-Tris 4% to 12% gradient acrylamide gels (Invitrogen) and transferred to nitrocellulose membrane (Invitrogen). Membranes were incubated in blocking buffer [50 mM Tris–HCl pH 7.4, 0.15 M NaCl, 0.25% bovine serum albumin (BSA), 0.05% (w/v) Tween-20, 0.05% NaN_3_ and 2% (w/v) fish skin gelatin] with the following primary antibodies: polyclonal sheep α-GFP antibody (MRC-PPU, 2–238, 1:10 000), rat α-VSG-2 (1:10 000), rabbit α-VSG-5 (1:10 000), mouse α-myc (Millipore, clone 4A6, 1:2000) and mouse α-EF1α (Millipore, CBP-KK1, 1:10 000). Detection was performed using IRDye 800CW donkey anti-goat (1:15 000) and IRDye 680RD donkey anti-mouse (1:10 000), respectively, in blocking buffer. The immunoblot was analysed on the LI-COR Odyssey Infrared Imaging System (LI-COR Biosciences).

### Metabolic labelling

For metabolic labelling, 10^7^ cells were collected by centrifugation (1000 × *g*, 10 min), washed and resuspended in methionine- and cytosine-depleted RPMI and dialyzed FBS (Thermo Fischer Scientific) for 15 min prior to labelling. Cells were then labelled with 50 μCi/ml ^35^S methionine (Perkin Elmer) for 5 min, washed in PBS, and resuspended in NuPAGE^®^ LDS sample buffer (Thermo Fisher) at 70°C for 10 min. Samples were run on 8%–12% gradient polyacrylamide gels (Life Technologies). Gels were vacuum dried using a 583 gel dryer (Bio-Rad). ^35^S incorporation was visualized on film after exposure at −80°C.

### Microscopy

Cells were fixed in 1% paraformaldehyde and attached to 12-well 5 mm slides (Thermo Scientific) by drying overnight for wide-field microscopy. For super-resolution microscopy, cells were attached to poly-L-lysine-treated coverslips, stained, and then mounted onto glass slides. Following rehydration in PBS for 5 min, cells were blocked with 50% FBS in PBS for 15 min. After two washes in PBS, cells were incubated in primary antibody for 1 h at RT: rat α-VSG-2 (1:10 000), rabbit α-VSG-5 (1:10 000), or mouse α-myc (1:2000). Following three washes in PBS, cells were incubated in secondary antibody for 1 h at RT: α-rat Alexa 488 (Life Technologies, 1:2000), α-rabbit Alexa 568 (Life Technologies, 1:2000), and α-mouse Alexa 488 (Life Technologies, 1:1000). Cells were washed three more times in PBS and mounted in Vectashield (Vector Laboratories) with DAPI (4′,6-diamidino-2-phenylindole) for wide-field microscopy or stained with 1 μg mL^–1^ DAPI for 10 min and mounted in Vectashield without DAPI for super-resolution microscopy. Cells were imaged as z-stacks (0.1–0.2 μm) at 63× magnification with oil immersion and a Zeiss Axiovert 200 M microscope with Zen Pro software (Zeiss) for wide-field microscopy or a Leica Stellaris 8 inverted confocal microscope equipped with Power HyD detectors and subjected to adaptive deconvolution using the integrated Leica LIGHTNING algorithm for super-resolution microscopy. Images were processed using Fiji v1.5.2e [36]. MCP^GFP^ fluorescence was directly visualized. For cell cycle analysis, cells were rehydrated in PBS and stained with DAPI (4′,6-diamidino-2-phenylindole) in Vectashield. For analysis of newly synthesized DNA, trypanosomes were collected by centrifugation at 1000 × *g* for 10 min, resuspended in thymidine-free HMI-11, and induced with tetracycline as required 24 h later. 5-ethynyl-2′-deoxyuridine (EdU) [37] incorporation was assessed as previously described [38], using the Click-iT EdU Alexa Fluor 555 imaging kit (Life Technologies), with some modifications: Briefly, cells were incubated with 150 μM EdU for 4–6 h. EdU was washed off, and cells were resuspended in 500 μl of media and mixed 1:1 with 2% formaldehyde in PBS for 1 h at 4°C. The formaldehyde was removed by washing cells twice in PBS and re-suspending in 1% BSA in dH_2_O before spreading on glass slides and drying overnight. Cells were rehydrated in PBS and the azide click chemistry reaction was performed as per the manufacturer’s instructions.

### Flow cytometry

For cell cycle analysis, cells were collected by centrifugation (1000 × *g*, 10 min) and washed in ice-cold PBS before being resuspended in 300 μl ice-cold PBS and fixed overnight with 700 μl methanol at −20°C. Fixed cells were washed twice with PBS before DNA staining with propidium iodide at 5 μg·ml^−1^, and RNA digestion with RNase A at 10 μg·ml^−1^ for 1 h at 37°C. For VSG detection, the primary antibodies were rat α-VSG-2 (1:10 000) and rabbit α-VSG-5 (1:10 000). Secondary antibodies were goat α-rat Alexa Fluor 647 (1:2000) and goat α-rabbit Alexa Fluor 488 (1:2000). Samples were analyzed on a BD FACSCanto (BD Biosciences), and data were visualized and processed using FlowJo software. Forward scatter area (FSC-A) versus forward scatter height (FSC-H) was used to exclude cell debris and aggregates. Cells exclusively expressing VSG-2 or VSG-5 were used to draw gates for cells expressing both VSGs.

### RNA-seq

RNA-seq was performed as described previously [39]. Briefly, 2T1, MCP*^VSG-2^*, or RNAi*^VSG-2^* cells were incubated with tetracycline for 0, 8, and 12 h. 1 × 10^8^ cells per condition were washed with PBS, and total RNA was extracted using an RNeasy kit (QIAGEN) according to the manufacturer’s instructions. Samples were sequenced in triplicate on a DNBSEQ-G400 platform (BGI, Hong Kong). Raw sequencing data were processed through a standardized pipeline. Initial quality control was performed using FastQC (https://www.bioinformatics.babraham.ac.uk/projects/fastqc/), followed by adapter trimming and quality filtering with Fastp (0.20.0) [40]. Processed reads were aligned to the *T*. *brucei* TREU927 reference genome v68 [41] supplemented with a set of 1200 bp truncated *VSGs* using Bowtie2 (2.3.5) [42] with ‘--very-sensitive-local’ parameters. The resulting alignments were processed with SAMtools (1.9) [43] for sorting and indexing, and PCR duplicates were marked using Picard MarkDuplicates (2.22.3) [44]. Read counts per coding sequence were quantified using featureCounts (1.6.4) [45] with parameters: -p (pair end) -B (both ends successfully aligned) -C (skip fragments that have their two ends aligned to different chromosomes) -M (count multi-mapping) -O (match overlapping features) -t CDS (count level) -g gene_id (summarization level). Genes with low counts were filtered out using edgeR [46]. Overall quality metrics for fastq files and alignments were aggregated and visualized with MultiQC [47]. Differential abundance analyses were carried out in R (3.6.1) with edgeR (3.28.0) using generalized linear models (GLM) and the correction factors for length and GC bias provided by the cqn package (1.32.0) [47]. Analysis of Gene Ontology enrichment was carried out using the TriTrypDB kinetoplastid informatics resource.

### Genome sequencing

Genomic DNA was isolated using a PureLink™ Genomic DNA Mini Kit (Invitrogen). Genome sequencing was performed using a DNBSEQ-G400 platform (MGI) and ∼60 million 100 bp paired-end reads were generated per sample (BGI, Hong Kong). Initial quality control of fastq files was performed using FastQC (https://www.bioinformatics.babraham.ac.uk/projects/fastqc/), followed by adapter trimming and quality filtering with Fastp (0.20.0) [40]. Processed reads were aligned to the reference genome 427_2018 (TriTrypDB v68) [48] using Bowtie2 (2.3.5) [42] with ‘--very-sensitive-local’ parameters. The resulting alignments were processed with SAMtools (1.9) [43] for sorting and indexing. Bam files were transformed to bedgraph track files using bamCoverage from DeepTools (3.5) [49] with --binSize 5000, --smoothLength 10 000, and --normalizeUsing RPKM. The linear coverage visualization was performed with a custom Python script.

### Proteomics

Mass spectrometry and proteomic analyses were performed as described previously [16]. MCP*^VSG-2^* or RNAi*^VSG-2^* cells were grown for 24 h with or without tetracycline. 5 × 10^7^ cells were washed in PBS and resuspended in 100 μL of a solution containing 5% SDS and 100 mM triethylammonium bicarbonate. Triplicate samples were submitted to the Fingerprints Proteomics Facility at the University of Dundee for analysis. Cell lysates were treated with 25 U of Benzonase (EMD Millipore, #70664) and sonicated for 2 min in a water bath sonicator. The protein concentration was then determined using the Micro BCA Protein Assay Kit (Thermo Fisher, #23235). From each lysate, a volume equivalent to 150 μg of protein was processed using S-Trap mini spin columns (Protifi, # CO2-mini-80) and following the default protocol. Briefly, the lysates were reduced and alkylated by the addition of 20 mM dithiothreitol (VWR, #M109-5G) and 40 mM iodoacetamide (Sigma–Aldrich, #I6125-10G), respectively. The proteins were then precipitated with the addition of 12% orthophosphoric acid (VWR, #20624.262) and 7× sample volume of Strap Binding Buffer [90% methanol (VWR, #83638.290) containing 100 mM TEAB (Sigma–Aldrich, #T7408-100ML)]. The acidified mixture was then placed into the spin columns, and after a centrifugation step, the columns were washed with Strap Binding Buffer. The proteins were digested overnight with the addition of trypsin (1:40, Thermo Fisher, #90057) at 37°C in a water-saturated atmosphere. Fresh trypsin (1:40) was then added and incubated for a further 6 h. The peptides were then eluted from the columns by adding 50 mM TEAB and centrifuging for 30 s; two more elution steps using 0.2% aqueous formic acid (Fisher Chemical, #A117-50) and 50% aqueous acetonitrile (VWR, #83640.290) containing 0.1% formic acid were also carried out. The peptides were then dried by vacuum centrifugation. The dried peptides were resuspended with 20 μl of 1% formic acid and were injected into a Q-Exactive Plus Mass Spectrometer (Thermo Fisher) for quality control assessment and quantification. Sample volumes equivalent to 1.5 μg of peptides were injected onto a nanoscale C18 reverse-phase chromatography system (Ultimate 3000 RSLC nano, Thermo Scientific) and electrosprayed into an Orbitrap Exploris 480 Mass Spectrometer (Thermo Fisher). For liquid chromatography, the following buffers were used: Buffer A [0.1% formic acid (FA, Fisher Scientific, #A117-50) in MilliQ water (v/v)] and Buffer B [80% acetonitrile (VWR, #83640.290, 0.1% FA in MilliQ water (v/v)]. Samples were loaded at 10 μl/min onto a trap column (100 μm × 2 cm, PepMap nanoViper C18 column, 5 μm, 100 Å, Thermo Scientific) equilibrated with 0.1% trifluoroacetic acid (TFA, Thermo Scientific, #85183). The trap column was washed for 3 min at the same flow rate with 0.1% TFA, then switched in-line with a Thermo Scientific resolving C18 column (75 μm × 50 cm, PepMap RSLC C18 column, 2 μm, 100 Å). Peptides were eluted from the column at a constant flow rate of 300 nl/min with a linear gradient from 3% buffer B to 6% buffer B in 5 min, then from 6% buffer B to 35% buffer B in 115 min, and finally from 35% buffer B to 80% buffer B within 7 min. The column was then washed with 80% buffer B for 4 min. Two blanks were run between each sample to reduce carryover. The column was kept at 50°C. The data were acquired using an easy spray source operated in positive mode with spray voltage at 2.40 kV and the ion transfer tube temperature at 250°C. The MS was operated in DIA mode. A scan cycle comprised a full MS scan (m/z range from 350 to 1650), with RF lens at 40%, AGC target set to custom, normalized AGC target at 300%, maximum injection time mode set to custom, maximum injection time at 20 ms, microscan set to 1, and source fragmentation disabled. MS survey scan was followed by MS/MS DIA scan events using the following parameters: Multiplex ions set to false, collision energy type set to normalized, HCD collision energies set to 25.5, 27, and 30%, orbitrap resolution 30 000, first mass 200, RF lens 40%, AGC target set to custom, normalized AGC target 3000%, microscan set to 1, and maximum injection time 55 ms. Loop control N, N (number of spectra set to 23). Data for both MS scan and MS/MS DIA scan events were acquired in profile mode. Analysis of the DIA data was carried out using Spectronaut (version 17.4.230317.55965, Biognosys, AG). The directDIA workflow, using the default settings (BGS Factory Settings) with the following modifications was used: decoy generation set to inverse, Protein LFQ Method set to QUANT 2.0 (SN Standard), Precursor Filtering set to Identified (Qvalue), Precursor Qvalue Cutoff and Protein Qvalue Cutoff (Experimental) set to 0.01, Precursor PEP Cutoff set to 0.01, Protein Qvalue Cutoff (Run) set to 0.01, and Protein PEP Cutoff set to 0.75. Cross-run normalization was selected, with Normalization Strategy set to Global (normalizing on the median). For the Pulsar search the settings were: maximum of two missed trypsin cleavages; PSM, protein, and peptide False Discovery Rate (FDR) levels set to 0.01; scanning ranges set to 300–1800 m/z and relative intensity (minimum) set to 5%; cysteine carbamidomethylation set as fixed modification; and acetylation (N-term), deamidation (asparagine, glutamine), dioxidation (methionine, tryptophan), glutamine to pyro-Glu, and oxidation of methionine set as variable modifications. Searches were made using a protein database of *T. brucei* TREU927 v51 obtained from TriTrypDB [41] combined with a predicted set of VSG proteins from the Lister 427 strain truncated at the first 400 AA.

### Differential protein abundance

Data analysis was performed using custom Python and R scripts, using the SciPy ecosystem of open-source software libraries [50]. A protein group pivot table was exported from the output of the Spectronaut analysis v15 (Biognosys). The protein groups identified as single hits were considered missing values. Protein groups with missing values in >50% of the samples were excluded from the analysis. The differential expression analysis was performed with limma v3.54 [51] after log_2_ transformation of the data. FDR values were computed with the toptable function in limma.

## Results

### Blocking *VSG* translation via MCP recruitment to the 5′-UTR

To probe roles for the *VSG* transcript that extend beyond encoding VSG, we sought a method to establish bloodstream-form trypanosomes in which *VSG* translation was specifically and conditionally blocked. For this purpose, we selected a system comprising the bacteriophage MS2 coat protein (MCP), which binds the MS2 RNA hairpin. This approach has been widely used in other organisms to block translation [35] and also in *T. brucei* to block GFP expression [52]. Morpholino oligonucleotides can also be used to block translation and have been used to block *VSG* translation [53], but this approach typically lacks conditional regulation and was not considered sufficiently penetrant for the studies we proposed.

**Figure 1.**
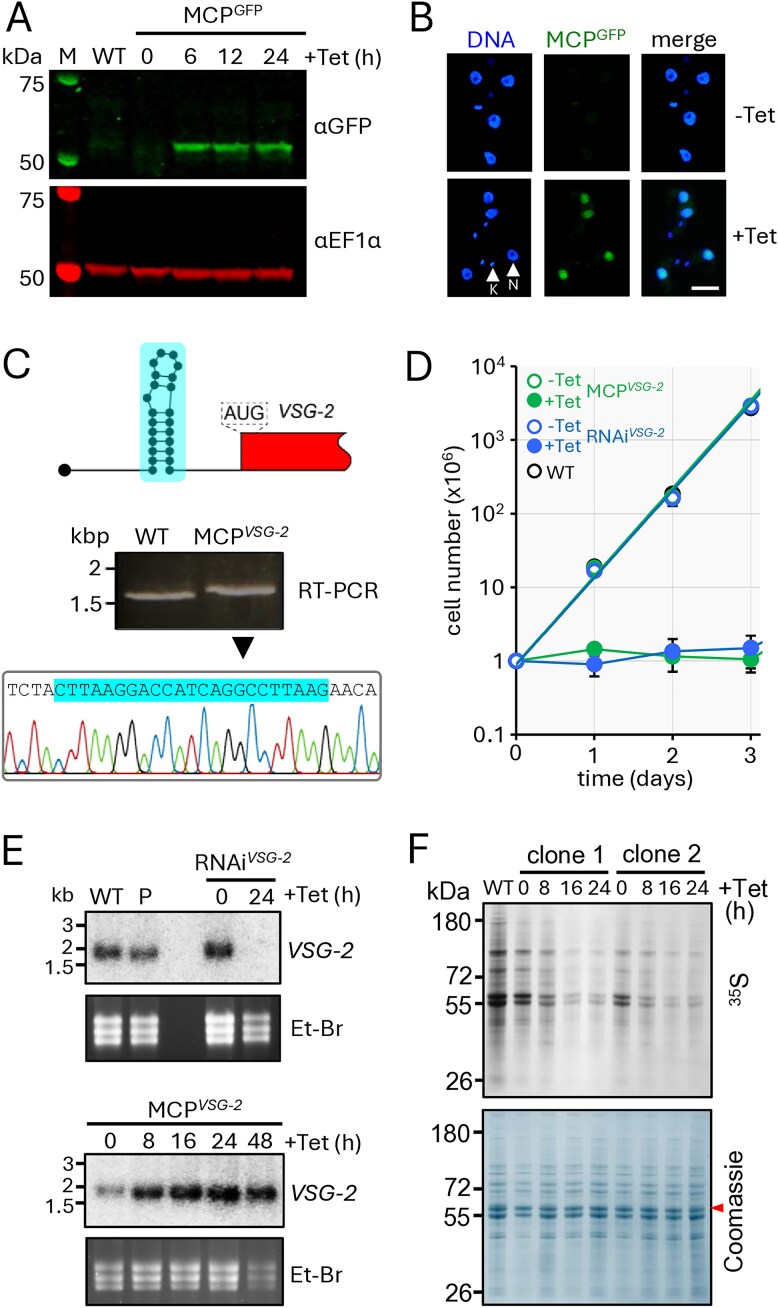
Blocking *VSG* translation via MCP recruitment to the 5′-UTR. (**A**) The protein blot shows tetracycline-inducible expression of MCP^GFP^ in *T. brucei*. EF1-α serves as a loading control. (**B**) Fluorescence microscopy reveals MCP^GFP^ in *T. brucei* nuclei following induction (+Tet, 24 h). Nuclear (N) and mitochondrial kinetoplast (K) DNA are indicated. (**C**) The schematic indicates an MCP-binding, MS2-RNA hairpin sequence in the *VSG-2* 5′-UTR in MCP*^VSG-2^* cells. The gel shows products obtained after *VSG-2*-specific RT-PCR, and the sequence trace shows incorporation of the MS2 hairpin sequence in MCP*^VSG-2^* cells. WT, wild-type. (**D**) Cumulative growth curves for wild-type cells and MCP*^VSG-2^* and RNAi*^VSG-2^* cells before and after induction. The data represent averages from two independent biological replicates. (**E**) The RNA blots show *VSG-2* mRNA abundance following knockdown in RNAi*^VSG-2^* cells or following MCP^GFP^ induction in MCP*^VSG-2^* cells. Ethidium bromide (Et-Br)-stained gels serve as loading controls. The data are representative of two independent biological replicates. WT, wild-type; P, parental strain. (**F**) Metabolic labelling with ^35^S methionine during MCP^GFP^ induction in MCP*^VSG-2^* cells; two biological replicates. The Coomassie-stained panel serves as a loading control; the red arrowhead indicates VSG-2.

Although *T. brucei* cells are diploid, a single *VSG* gene is expressed at a hemizygous sub-telomeric locus [54]. To block translation of the single expressed *VSG-2* gene, we first assembled a strain for tetracycline-inducible expression of MCP^GFP^, with an *N*-terminal La nuclear localization signal [33] and with GFP fused to the C-terminus. MCP^GFP^ expression was tetracycline-inducible (Fig. 1A), and the protein accumulated in the nucleus, as expected (Fig. 1B). We next generated strains containing both inducible MCP^GFP^ and a single MS2 ‘hairpin sequence’ in the 5′-UTR of the *VSG-2* gene (Fig. 1C). Correct integration of the hairpin sequence in the *VSG-2* gene and incorporation into *trans*-spliced mRNA in these ‘MCP*^VSG-2^*’ cells was confirmed by reverse transcription polymerase chain reaction (RT-PCR) and sequencing (Fig. 1C).

For comparison with MCP-based translation blockade, we generated RNAi*^VSG-2^* strains containing an inducible *VSG-2* knockdown cassette. We then compared growth of wild-type cells, and the MCP*^VSG-2^* and RNAi*^VSG-2^* strains under non-inducing and inducing conditions. All strains displayed comparable exponential growth under non-inducing conditions (Fig. 1D), indicating both that MCP^GFP^ expression is tightly regulated and that the modified *VSG-2* mRNA had no detectable deleterious impact on fitness in the MCP*^VSG-2^* strains prior to MCP^GFP^ induction. Following induction of knockdown, the RNAi*^VSG-2^* strains displayed a severe growth defect under inducing conditions, as expected [55], and the MCP*^VSG-2^* strains displayed a similar profile following induction of MCP^GFP^ expression (Fig. 1D).

Using RNA blotting, we next assessed *VSG-2* transcript abundance following induction of *VSG-2* knockdown or translation blockade. This analysis confirmed *VSG-2* mRNA knockdown in RNAi*^VSG-2^* cells as anticipated (Fig. 1E). In contrast, *VSG-2* mRNA was stabilized following induction of MCP^GFP^ expression in MCP*^VSG-2^* cells; indeed, *VSG-2* mRNA abundance was increased in these cells (Fig. 1E), perhaps due to increased m^6^A modification and/or CFB2-binding [17, 27].

To determine whether *VSG* translation was indeed blocked in MCP*^VSG-2^* cells, we analysed protein synthesis under inducing conditions. Metabolic labelling of newly synthesized proteins with ^35^S-methionine revealed reduced global translation in two independent MCP*^VSG-2^* strains (Fig. 1F) and quantification revealed that protein synthesis was reduced by 90% after 16 h. Thus, VSG translation blockade brings about global translation arrest, as also reported following *VSG* RNAi [56]. We concluded that MCP^GFP^-dependent *VSG* translation blockade, like *VSG* transcript knockdown, triggered global translation arrest and a severe growth defect. VSG translation blockade therefore phenocopied defects associated with loss of VSG expression, while maintaining the cellular pool of *VSG* mRNA. The major difference in *VSG-2* mRNA abundance observed in MCP*^VSG-2^* and RNAi*^VSG-2^* strains, therefore, presented an opportunity to investigate specific roles of the *VSG* mRNA.

### Native *VSG* exclusion is sustained following VSG-2 perturbation

**Figure 2.**
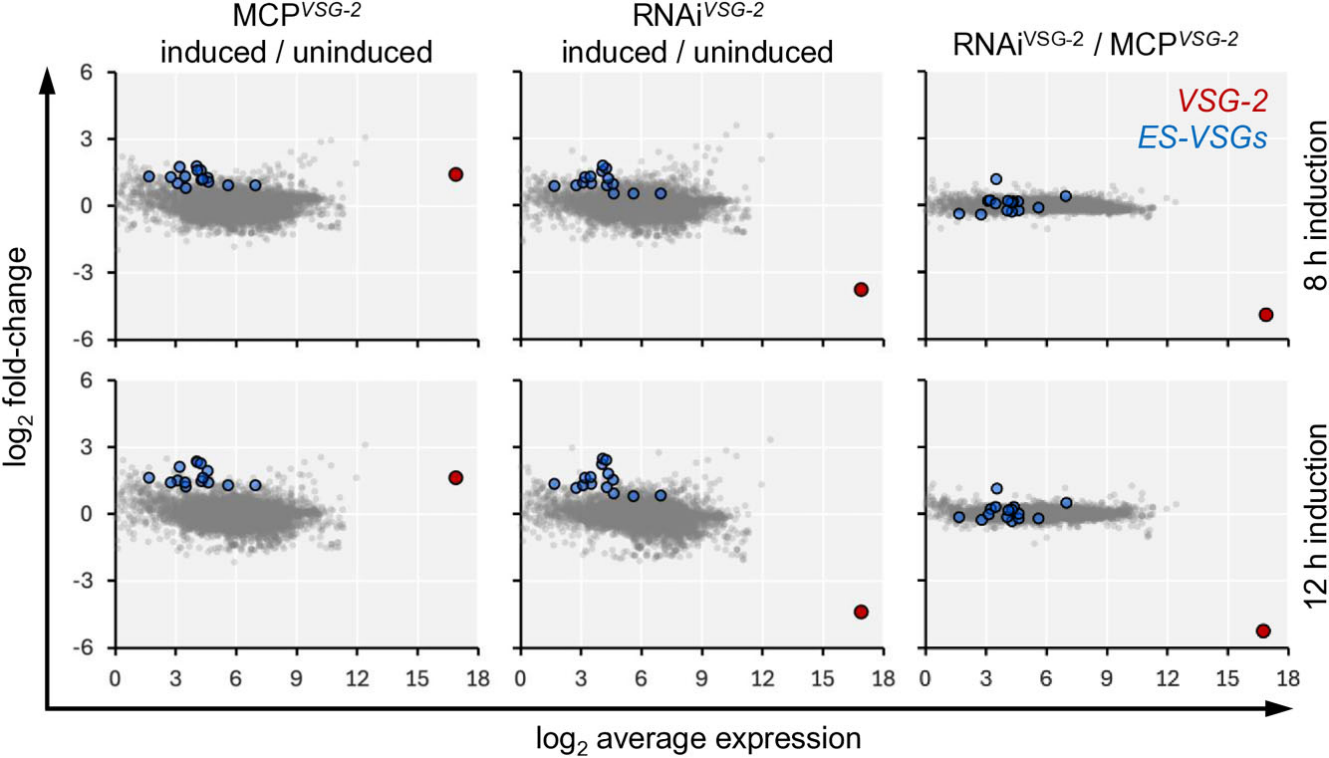
Native *VSG* exclusion is sustained following VSG-2 perturbation. RNA-seq analysis following induction of translation blockade in MCP*^VSG-2^* cells or *VSG-2* mRNA knockdown in RNAi*^VSG-2^* cells for either 8 h (upper panels) or 12 h (lower panels). *VSG-2* and other excluded expression-site-associated *VSG* transcripts are highlighted. The data represent averages from three independent technical replicates in each case. *n* = 8458.

To investigate specific non-coding roles for the *VSG* transcript, we first used RNA-seq to assess the transcriptomes following either translation blockade or *VSG-2* knockdown. *VSG-2* transcript abundance was not significantly different in these strains prior to induction (FDR = 0.5). Following induction of each perturbation for either 8 h or 12 h, we observed increased *VSG-2* transcript abundance following translation blockade in MCP*^VSG-2^* cells and *VSG-2* knockdown in RNAi*^VSG-2^* cells (Fig. 2), supporting the results from RNA blotting above (Fig. 1E). Translation blockade increased the abundance by >2-fold after 8 h (FDR = 1.2e^−^6), confirming that translation blockade following recruitment of the MCP does indeed stabilize the *VSG-2* transcript, while knockdown reduced *VSG-2* transcript by >90% after 8 h (FDR = 3e^−^13). Although VSG feedback to regulate *VSG* transcript abundance has been reported previously [57], the increase in abundance of the already super-abundant *VSG-2* transcript is quite remarkable, given a starting point of ∼10% of total cell mRNA [9]. We concluded that *VSG-2* transcript abundance was 24- or 39-fold lower in RNAi*^VSG-2^* cells than in MCP*^VSG-2^* cells following 8 or 12 h of induction, respectively.

Despite the difference in *VSG-2* transcript abundance, other telomeric *VSGs* were not substantially derepressed in either of these strains (Fig. 2). Among fifteen expression-site-associated *VSGs* detected, we saw <3-fold average de-repression in MCP*^VSG-2^* and RNAi*^VSG-2^* strains, with these *VSGs* remaining >2500-fold lower in abundance on average than the unperturbed active *VSG-2* transcript. Indeed, with the notable exception of *VSG-2*, the transcriptomes of MCP*^VSG-2^* and RNAi*^VSG-2^* strains were otherwise similar following 12 h of induction (Fig. 2, right-hand panels). Thus, neither blocking translation by recruiting MCP nor knockdown of the active *VSG* transcript using RNA interference substantially impacted the expression of established excluded *VSGs*. We conclude that *VSG* exclusion was sustained when the dominant *VSG* transcript was depleted for 12 h and by >15-fold.

### A *VSG* transgene evades exclusion when the *VSG-2* transcript is depleted

**Figure 3.**
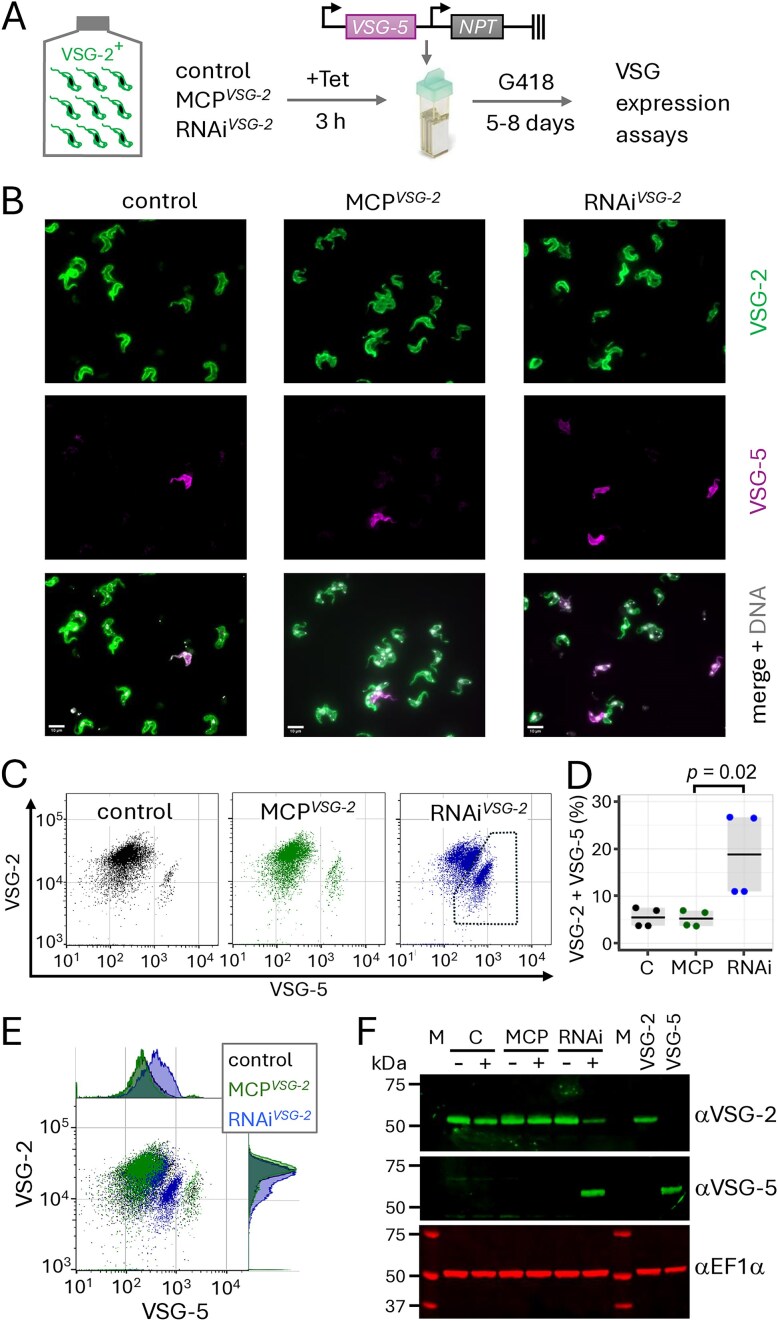
A *VSG* transgene evades exclusion when *VSG-2* is depleted. (**A**) The schematic illustrates the experimental procedure. MCP*^VSG-2^* and RNAi*^VSG-2^* strains were induced with tetracycline (Tet) for 3 h, Tet was removed, and cells were transfected with the *VSG-5* reporter; the arrows indicate *rDNA* promoters, and the symbol on the right indicates a *de novo* telomere. Cells were then selected for *NPT* expression using G418 and *VSG* expression was assessed after 5–8 days. (**B**) Representative fluorescence microscopy images reveal VSG-2 and VSG-5 expression. Scale bars, 10 μm. (**C**) Flow cytometry analysis of VSG-2 and VSG-5 expression. *n* = 10 000 cells in each case. (**D**) Quantification of cells expressing both VSG-2 and VSG-5 according to the gated region indicated in panel (C) (right-hand panel). The data are from two independent biological replicates and two technical replicates. Horizontal lines indicate mean values. The *P*-value was calculated using a two-sided *t*-test. (**E**) Data from panel (C) were combined to compare bulk population profiles for each strain. (**F**) The protein blot shows VSG-2 and VSG-5 expression in control cells (C) and MCP*^VSG-2^* (MCP) and RNAi*^VSG-2^* (RNAi) cells before (−) and after (+) introduction of the *VSG-5* transgene, according to the protocol shown in panel (A). EF1-α serves as a loading control. Cells exclusively expressing VSG-2 or VSG-5 are included as controls. M, molecular weight markers.

The results above indicated that native *VSG* exclusion was maintained when *VSG-2* translation was blocked or when *VSG-2* mRNA was depleted. Next, we asked whether *VSG* transcripts compete for establishment of the dominant active state. To address this question, we used an expression assay with a *VSG* transgene that is known to be subject to exclusion [19]. MCP*^VSG-2^* and RNAi*^VSG-2^* strains were transiently induced for 3 h prior to washing and delivery of the *VSG-5* transgene, which, when integrated into the genome, is transcribed from an *rDNA* promoter adjacent to a *de novo* telomere [19]; *rDNA* promoters are distinct from *VSG* expression site promoters but they can replace *VSG* promoters and are subject to the exclusion mechanism [58]. Five days following delivery of the transgene, the resulting cells were stained for cell-surface VSG-2 and VSG-5 expression and were assessed using immunofluorescence microscopy and flow cytometry (Fig. 3A).

The *VSG-5* transgene behaved as expected in the control cells [19], with only 5.5% of cells on average expressing the transgene, and the results were very similar (5.2%) following transient *VSG*-2 translation blockade in MCP*^VSG-2^* cells (Fig. 3B and C, left-hand and middle panels; Fig. 3D and E). In contrast, the VSG-5 signal was increased for a significantly higher proportion of cells (18.8%) following transient *VSG*-2 knockdown (Fig. 3B and C, right-hand panels; Fig. 3D and E). Indeed, the bulk population of RNAi*^VSG-2^* cells displayed increased VSG-5 expression relative to control and MCP*^VSG-2^* cells (Fig. 3E); similar results were obtained in three separate experiments conducted by two independent investigators. To confirm this finding, we repeated the process outlined in Fig. 3A and ran a further orthogonal protein blotting assay. This again revealed increased *VSG-5* transgene expression specifically following transient *VSG-2* knockdown (Fig. 3F). We concluded that a transgenic *VSG-5* reporter evaded exclusion when delivered under transient *VSG-2* knockdown. These results suggest that the establishment of monogenic *VSG* expression is driven by competing *VSG* transcripts.

### *VSG* transcript perturbation impacts CFB2 abundance

**Figure 4.**
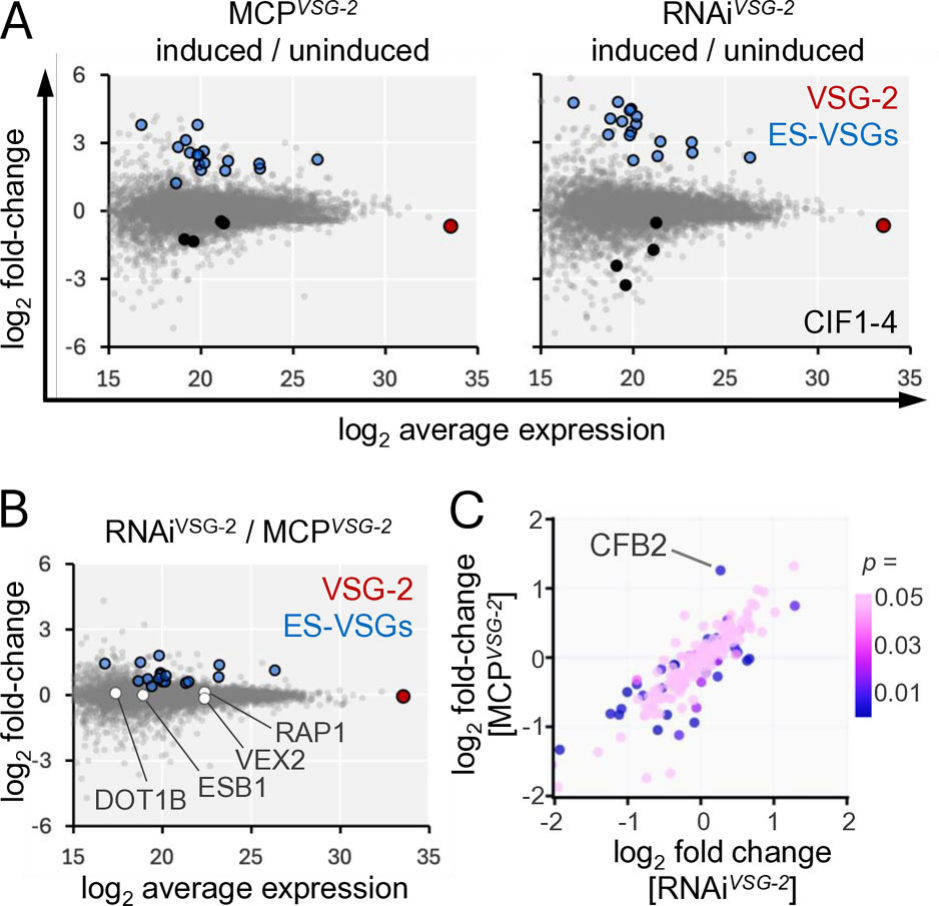
*VSG* transcript perturbation impacts CFB2 abundance. (**A**) Proteomics analysis following induction of translation blockade in MCP*^VSG-2^* cells or *VSG-2* mRNA knockdown in RNAi*^VSG-2^* cells for 24 h. *VSG-2*, other excluded expression-site-associated *VSG* transcripts, and CIF1-4 are highlighted. The data represent averages from three independent technical replicates in each case. *n* = 5552. (**B**) As in panel (A), but showing a comparison between *VSG-2* mRNA knockdown in RNAi*^VSG-2^* cells and translation blockade in MCP*^VSG-2^* cells. *VSG* regulatory factors are also highlighted. (**C**) Proteomics analysis of all detected proteins associated with the ‘mRNA-binding’ Gene Ontology term (*n* = 181).

We next wondered whether phenotypes associated with *VSG* perturbation might be associated with specific changes in VSG or regulatory factor abundance, and we used quantitative proteomic analysis to address this question. Consistent with failure to synthesize new VSG, we observed similar (>30%) and significant VSG-2 depletion following 24 h of either *VSG-2* translation blockade (FDR = 9e^−^6) or knockdown (FDR = 1e^−^5) (Fig. 4A); depletion was likely limited because VSG has a half-life of approx. 30 h [59]. As observed using transcriptome analysis above (Fig. 2), other expression-site-associated *VSGs* were moderately derepressed in both the MCP*^VSG-2^* and RNAi*^VSG-2^* strains (Fig. 4A); although those VSGs detected remained >500-fold lower in abundance on average than unperturbed VSG-2. Notably though, we did observe significantly higher VSG derepression following knockdown (Fig. 4B).

The proteomes of MCP*^VSG-2^* and RNAi*^VSG-2^* strains were otherwise similarly perturbed following induction (Fig. 4B). For example, Gene Ontology analysis of the 200 proteins most significantly reduced in abundance in each case (Supplementary Data 1) revealed enrichment for ‘mRNA-binding’ (RNAi*^VSG-2^*, *P* = 1.1e^−^9; MCP*^VSG-2^ P* = 3.8e^−^8), ‘ribosome biogenesis’ (RNAi*^VSG-2^*, *P* = 1.1e^−^5; MCP*^VSG-2^ P* = 3.7e^−^17), and ‘cleavage furrow’ (RNAi*^VSG-2^*, *P* = 1.4e^−^4; MCP*^VSG-2^ P* = 2.7e^−^3), consistent with the translation blockade and cytokinesis arrest phenotypes described earlier. Indeed, all four components of the cytokinesis initiation factor (CIF1-4) [60] were significantly reduced in abundance in both strains (Fig. 4A and Supplementary Data 1). We next assessed several known *VSG* expression regulators for differences in abundance following either perturbation. This analysis revealed no significant differences for ESB1, VEX2, RAP1, or DOT1B (Fig. 4B), but did reveal a significant difference for the cyclin-like F-box protein, CFB2. Indeed, analysis of all proteins associated with the ‘mRNA-binding’ Gene Ontology term (*n* = 181) highlighted CFB2 (Fig. 4C), which was increased in abundance (>2-fold) following translation blockade (FDR = 4e^−^5), consistent with binding and stabilization of the *VSG* transcript [17], but was not significantly changed following *VSG-2* knockdown (FDR > 0.1).

### An additional round of DNA replication in the presence of the *VSG* transcript

**Figure 5.**
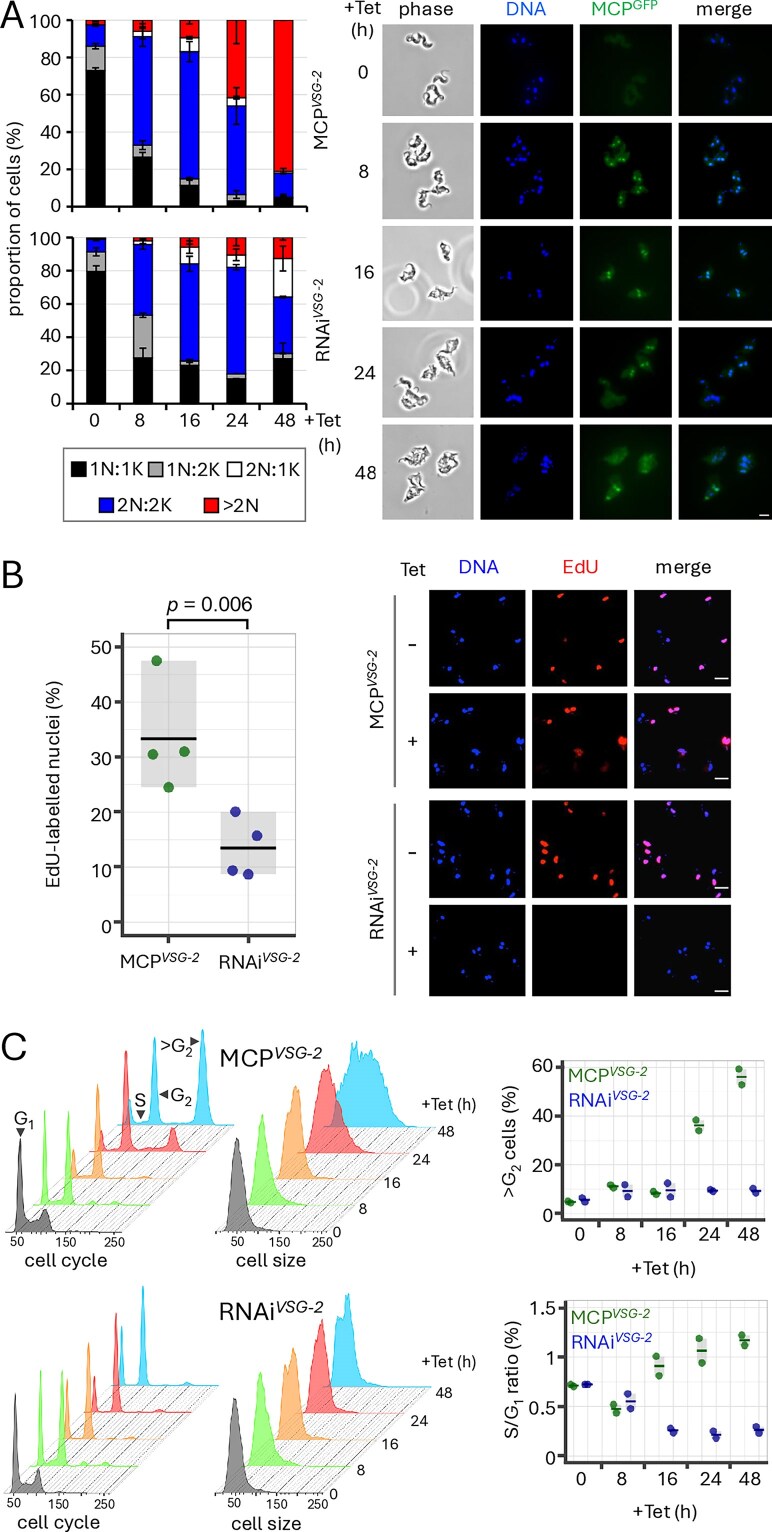
An additional round of DNA replication in the presence of the *VSG* transcript. (**A**) Microscopy-based quantification of the number of nuclei and kinetoplasts per cell following induction in MCP*^VSG-2^* cells (upper panel) or RNAi*^VSG-2^* cells (lower panel). The data represent averages from two independent biological replicates. Representative fluorescence microscopy images of MCP*^VSG-2^* cells are shown on the right. 2N cells (white and blue categories) are post-mitotic. DNA was stained with DAPI, while GFP reveals the expression and location of MCP^GFP^. Scale bar, 5 μm. (**B**) Microscopy-based quantification of the proportion of EdU-labelled nuclei in MCP*^VSG-2^* or RNAi*^VSG-2^* cells following induction for 16 h. The data are from two independent biological replicates assessed in duplicate. Horizontal lines indicate mean values. The *P*-value was calculated using a two-sided *t*-test. Representative fluorescence microscopy images are shown on the right. DNA was stained with DAPI. Scale bars, 10 μm. (**C**) Flow cytometry analysis following MCP^GFP^ induction in MCP*^VSG-2^* cells, or *VSG-2* knockdown in RNAi*^VSG-2^* cells. Representative histograms indicate DNA content based on propidium iodide staining (left-hand ‘cell cycle’ panels) or relative cell size based on side scatter (right-hand ‘cell size’ panels). The plots on the right indicate proportions of cells with a > G_2_ DNA content and the S phase to G_1_ ratios. The data are from two independent biological replicates. Horizontal lines indicate mean values.

Although it is known that *VSG* knockdown leads to S phase, mitosis, and cytokinesis arrest [55, 61], potential roles for the *VSG* transcript in controlling progression through the cell cycle have not been explored. Our MCP*^VSG-2^* and RNAi*^VSG-2^* strains presented an opportunity to explore such roles. Indeed, we were particularly interested in exploring connections between *VSG* transcript and DNA replication control since the transcript binds CFB2, which also interacts with the S-phase kinase associate protein, SKP1 [17]. To assess the impact of translation blockade or *VSG-2* knockdown on cell cycle progression, we used DNA staining and microscopy to visualize nuclear and mitochondrial (kinetoplast) DNA. This analysis revealed a similar dramatic increase in post-mitotic (2N:2K) cells after only 8 h of induction in both cases (Fig. 5A). A striking difference, however, was the emergence of cells with supernumerary (>2) nuclei, specifically following translation blockade, which comprised >40% of the population 24 h after induction and >80% at 48 h (Fig. 5A). These results are consistent with the view that efficient VSG trafficking to the cell surface is required for cytokinesis [55] and also now indicate that retention of the *VSG* transcript allows an additional round of mitosis. Notably, Ridewood *et al.* also observed 29% of cells with supernumerary nuclei when *VSG* was expressed at sub-optimal levels [61].

To explore DNA replication status, we labelled cells for 6 h with 5-ethynyl-2′-deoxyuridine (EdU) and quantified the proportion of cells replicating their nuclear genome by fluorescence microscopy. DNA in control cells, and in uninduced MCP*^VSG-2^* and RNAi*^VSG-2^* cells (Fig. 5B) was efficiently labelled with EdU (94, 98, and 97% of cells, respectively). Although both MCP*^VSG-2^* and RNAi*^VSG-2^* strains displayed decreased EdU labelling following growth under inducing conditions, labelling was significantly higher (*P*= .006) in the MCP*^VSG-2^* population, than in the RNAi*^VSG-2^* population (Fig. 5B). Finally, we used flow cytometry to quantify DNA content. Consistent with the DNA staining, EdU labelling, and microscopy analysis (Fig. 5A and B), we observed a specific increase in cells with a > G_2_-phase DNA content, and an increase in cell size, in the induced MCP*^VSG-2^* cells (Fig. 5C), indicating endoreduplication in cells that retain the *VSG* transcript. Although pre-cytokinesis arrest progressively diminished the proportion of G_1_ cells in both cases, the S phase/G_1_ ratio was significantly different 16 h post-induction (*P* = .02, two-sided *t*-test) and thereafter, being increased in the induced MCP*^VSG-2^* cells and decreased in induced RNAi*^VSG-2^* cells (Fig. 5C). Thus, we observed an additional round of DNA replication and mitosis in the presence of the *VSG* transcript that was not observed following loss of the *VSG* transcript.

### A second nuclear VEX2 focus in cells expressing a second *VSG*

**Figure 6.**
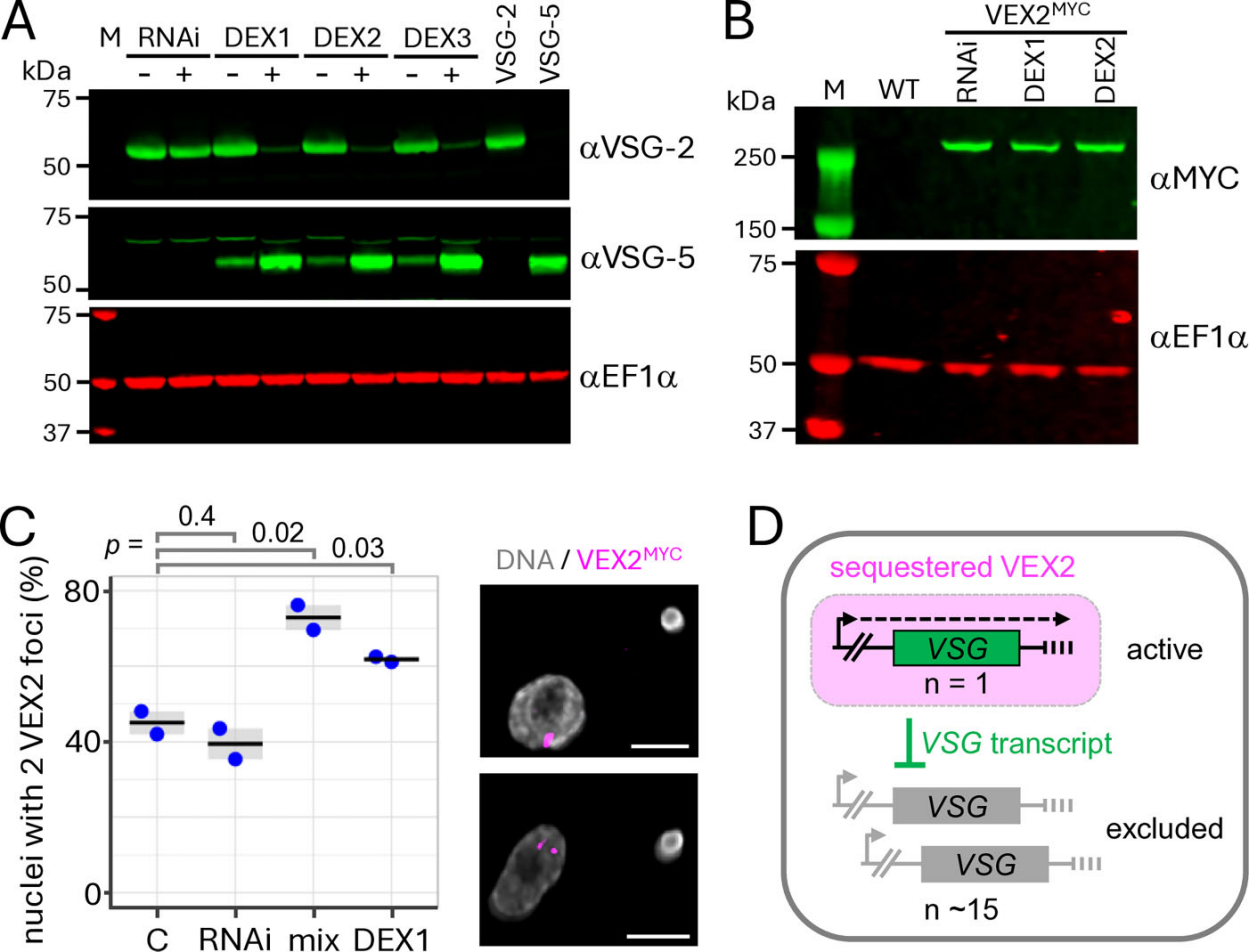
A second nuclear VEX2 focus in cells expressing a second *VSG*. (**A**) The protein blot shows VSG expression in RNAi*^VSG-2^* cells (RNAi) and double-expressing (DEX) RNAi*^VSG-2^* sub-clones generated according to the protocol shown in Fig. 3A. *VSG-2* knockdown was (re)induced (+) in each clone for 72 h. EF1-α serves as a loading control. Cells exclusively expressing VSG-2 or VSG-5 are included as controls. M, molecular weight markers. (**B**) The protein blot shows VEX2^MYC^ expression in RNAi*^VSG-2^* cells (RNAi) and in DEX cells from panel (A). Wild-type (WT) cells are included as a negative control. EF1-α serves as a loading control. M, molecular weight markers. (**C**) The plot shows the proportion of G_1_ nuclei with two VEX2^MYC^ foci in otherwise wild-type control cells (C), in RNAi*^VSG-2^* cells (RNAi), in an uncloned RNAi*^VSG-2^* population transformed with the *VSG-5* transgene according to the protocol shown in Fig. 3A (mix), and in the DEX1 clone; G_1_ nuclei are defined by a single rounded kinetoplast (*n* > 80). The vast majority of other G_1_ nuclei had a single VEX2 focus. The data are from counts carried out by two of us, with samples blinded in one case. Horizontal lines indicate mean values. The *P*-values were calculated using two-sided *t*-tests. The super-resolution fluorescence microscopy images show representative examples of VEX2^MYC^ foci (magenta) in RNAi*^VSG-2^* (upper panel) and DEX1 cells (lower panel). DNA was stained with DAPI (grey). Scale bars: 2 μm. (**D**) A winner-takes-all, RNA- and VEX2-mediated symmetry-breaking model for *VSG* allelic exclusion. The outer box represents the nucleus. *VSG* transcription and VEX2 recruitment are mutually reinforcing. The *VSG* transcript is a cncRNA that acts as a *trans*-repressor for competing *VSGs*.

Turning our attention back to allelic competition, we asked whether nuclear *VSG* expression compartments might be compromised in cells that expressed both *VSG-2* and the *VSG-5* transgene. VEX2 is required to maintain *VSG* exclusion, and is a putative helicase that forms an inter-chromosomal protein bridge connecting the *VSG* transcription and (VEX1-associated) splicing compartments [12, 13]. We considered a hypothesis whereby *VSG* transcription promotes RNA-mediated symmetry breaking and changes in nuclear architecture, as proposed for olfactory receptor exclusion [62]. To further explore this hypothesis, we MYC-epitope-tagged a native copy of *VEX2* in RNAi*^VSG-2^* cells and again followed the procedure detailed in Fig. 3A. We subcloned the resulting populations and assessed VSG-2 and VSG-5 expression by protein blotting and microscopy. *VSG-5* transgene expression was again detected following transient *VSG-2* knockdown, indicating relatively stable expression following escape from exclusion (Fig. 6A and Supplementary Fig. S1A); doubling times for these cells were 7.5 ± 0.4 h, compared to 6 h for RNAi*^VSG-2^* cells. Notably, when we re-induced *VSG-2* knockdown, VSG-2 expression was substantially reduced and VSG-5 expression was substantially increased in all three clones (Fig. 6A), suggesting continued competition in these double VSG-expressing cells, at the cell surface, and perhaps also in the nucleus.

To explore impacts on nuclear architecture, we assessed VEX2 localization in cells lacking the *VSG-5* transgene and in cells expressing both VSG-2 and VSG-5. VEX2^MYC^ was expressed at similar levels in these cells (Fig. 6B), but the proportion of nuclei with a second VEX2^MYC^ focus increased by ∼20% (*P* = .03) in the double VSG-expressing cells (Fig. 6C); and we saw a similar difference between RNAi*^VSG-2^* cells lacking the *VSG-5* transgene, and the population of RNAi*^VSG-2^* cells transformed with the *VSG-5* transgene and analysed prior to subcloning. It remains to be determined whether additional VEX2 foci reflect sites of transgenic *VSG-5* expression, but the results suggest *VSG* expression at two distinct sites, unlike colocalization of two active *VSGs* at the same nuclear site, as described by Budzak *et al.* [7]. Finally, we sequenced the genomes of two double VSG-expressing clones to determine where the *VSG-5* transgene had integrated, revealing integration on chromosome 7 in both cases (Supplementary Fig. S1B); *VSG-2* is on chromosome 6a. These results are consistent with an RNA-mediated symmetry-breaking model (Fig. 6D), as also recently proposed for monogenic olfactory receptor choice [62].

## Discussion

To probe roles of the *VSG* transcript that extend beyond encoding VSG, we assembled and compared strains in which *VSG* translation was conditionally blocked, with strains in which the *VSG* transcript was conditionally knocked down. The *VSG* transcript was found to be required to establish silencing of a *VSG* transgene and was also linked to DNA replication control. We suggest that *VSG* transcripts are coding and non-coding RNAs (cncRNAs) with a nuclear function, driving competition among *VSG* alleles to establish dominance and exclusion.

In terms of cell cycle controls, blocking VSG synthesis triggers a pre-cytokinesis arrest [53, 55] and a global translation arrest [56] in bloodstream form *T. brucei*, and similar phenotypes are observed following knockdown of other factors with roles in VSG coat maintenance: PFR2 (paraflagellar rod 2), actin, and clathrin [63–65], for example. We now confirm that the presence of the *VSG* transcript fails to rescue these phenotypes. On the other hand, our findings suggest that the *VSG* transcript can promote DNA replication, perhaps via a mechanism involving binding the bloodstream form-specific cyclin-like F-box protein, CFB2 [16, 17]. Metazoan Y RNAs have also been implicated in promoting DNA replication [66, 67], while embryonic stem cells can proliferate independent of G_1_ cyclins [68]. In addition, mammalian long non-coding RNAs regulate the expression of cyclins and CDKs [69]; the *gadd6* lncRNA regulates the G_1_/S checkpoint by promoting the degradation of the *Cdk6* transcript [70], for example.

There are remarkable differences in cell cycle controls operating in bloodstream and insect-form *T. brucei*. CRK1 and CRK2 promote the G_1_/S transition in insect-form cells but are not required for this purpose in bloodstream-form cells [71], for example. DNA replication continues in bloodstream-form cells, but not in insect-form cells, following knockdown of the CRK3 partner, CYC6 [72], or the chromosomal passenger protein, aurora-B kinase, AUK1 [73]. Knockdown of flagellar function [74], actin [65], or glycophosphatidylinositol anchor biosynthesis [75], all of which perturb VSG coat maintenance in bloodstream-form *T. brucei*, also result in the accumulation of multi-nucleated cells specifically in the bloodstream-form. Our findings, now connecting the *VSG* transcript to an additional round of DNA replication and mitosis, may explain these bloodstream-form-specific endoreduplication phenotypes, and the absence of such a phenotype following *VSG* knockdown [55, 61]. We suggest that novel checkpoint controls operate in bloodstream-form trypanosomes whereby cells monitor whether the demand for *VSG* mRNA and protein have been satisfied prior to committing to S phase or cytokinesis, respectively. Indeed, S phase may be a key point in the cell cycle when the competition among *VSG* transcripts operates.

Our main focus here was to explore RNA-mediated silencing in the *VSG* exclusion system. Indeed, we previously suggested a role for RNA-based, homology-dependent repression in *VSG* allelic exclusion, since reporters lacking *VSG*-associated sequences but with other common sequences were also subject to exclusion [19]. Antisense RNAs that modulate translation [76] or long non-coding RNAs that regulate differentiation [77, 78] have been reported in *T. brucei*. RNA interference also operates, but argonaut 1 (AGO1) knockout had no impact on *VSG* exclusion [79], suggesting an RNAi-independent mechanism. Specific or homologous sequences in the *VSG* transcript may be involved in the non-coding functions we propose here, and the 3′-UTR incorporates a highly conserved 16-mer motif immediately preceding the polyadenylation site. This motif is thought to bind CFB2 [17] and RAP1 [20], and to be required for m^6^A modification [27]. Indeed, the transgene we used in our assays incorporated this 16-mer within a 76 bp 3′-UTR that is identical to the native active *VSG-2* sequence [19]. Our favoured model involves competition for a limiting *trans*-activator, accompanied by negative control by *VSG* RNA at competing *VSG* loci (Fig. 6D), via R-loop formation [20, 80], for example. Recent findings reported by others are consistent with this model; specifically, transcription of a second *VSG* with a mutated 16-mer failed to silence the active *VSG* [81].

To interpret our findings, it is important to consider distinct mechanisms contributing to establishment and maintenance in the *VSG* exclusion system. We suggest that establishment of exclusion involves a competition among *VSG* transcripts for binding and sequestration of chromatin-associated RNA-binding proteins, such as VEX2 [18], RAP1 [20], or ESB1 [14]. Indeed, we observe a second nuclear VEX2 focus in cells expressing a second *VSG* following transient *VSG* transcript knockdown. Both establishment and maintenance of the active and silent states likely also require the action of additional chromatin-associated factors, including those factors enriched at the active transcription and splicing compartment [12–14]. In addition, the DOT1B histone methyltransferase is required to rapidly establish the excluded state [23, 24], while maintenance of exclusion is compromised when histones are depleted [25]. The chromatin chaperone, CAF-1 maintains the VEX complex at the active site [18, 25], while cohesin promotes inheritance of the active *VSG* during S phase [26]. Modification of the *VSG* transcript with m^6^A in the polyA-tail [27] may also promote chromatin accessibility [82].

Although multiple factors likely participate in establishing and maintaining active and silent *VSGs*, our findings suggest a central role for the *VSG* transcript. We show that the *VSG* transcript is a cncRNA, analogous to bi-functional cncRNAs that control developmental processes in vertebrates and plants [83]. We propose a ‘winner-takes-all’ model whereby the *VSG* cncRNA competes for sequestration of transactivators, such as ESB1 [14], and VSG exclusion factors [13], thereby modifying nuclear architecture to increase its own transcription and establish transcriptional dominance (Fig. 6D). Since we observe a profound collapse in *VSG* exclusion following VEX2 knockdown [13], we further suggest that VEX2 participates, with the *VSG* cncRNA, in negative control over distance to maintain dominance. Notably, competition among transcripts has also been proposed to drive olfactory receptor allelic exclusion and symmetry breaking in mammals [62, 84], while ncRNA also impacts *var* gene exclusion in malaria parasites [85]. We conclude that the *VSG* transcript is a cncRNA that inhibits the expression of its competitors.

## Supplementary Material

gkaf1011_Supplemental_Files

## Data Availability

RNA sequencing data and genome sequencing data have been deposited in the European Nucleotide Archive, www.ebi.ac.uk/ena (BioProject ID: PRJNA1216521). The mass spectrometry proteomics data have been deposited at the PRIDE repository (Dataset identifier PXD060524).

## Notes

Present address: Quantum-Si France, 38 Rue De Berri 75008 Paris, France
