# Supplementary material for: A non-coding role for trypanosome VSG transcripts in allelic exclusion
Source: Nucleic Acids Res. 2025 Oct 21;53(19):gkaf1011. doi: 10.1093/nar/gkaf1011 (PMC12539627; doi:10.1093/nar/gkaf1011)
Supplement: gkaf1011_Supplemental_Files [file gkaf1011_supplemental_files.zip › Fig. S1.pdf]

A

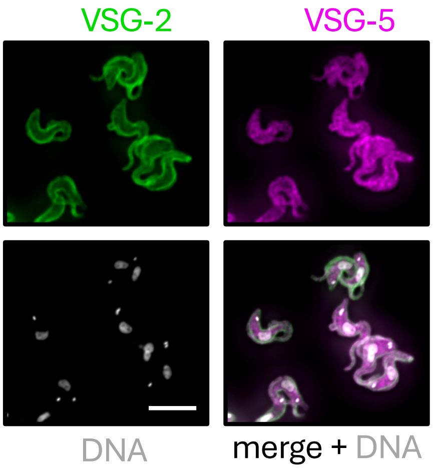

B

	length	*rDNA* promoter	lost	lost (%)
Chr1_core_Tb427v10	844108	620950	223158	26.4
Chr3_core_Tb427v10	1459197	812483	646714	44.3
Chr7_core_Tb427v10	2289182	1947579	341603	14.9

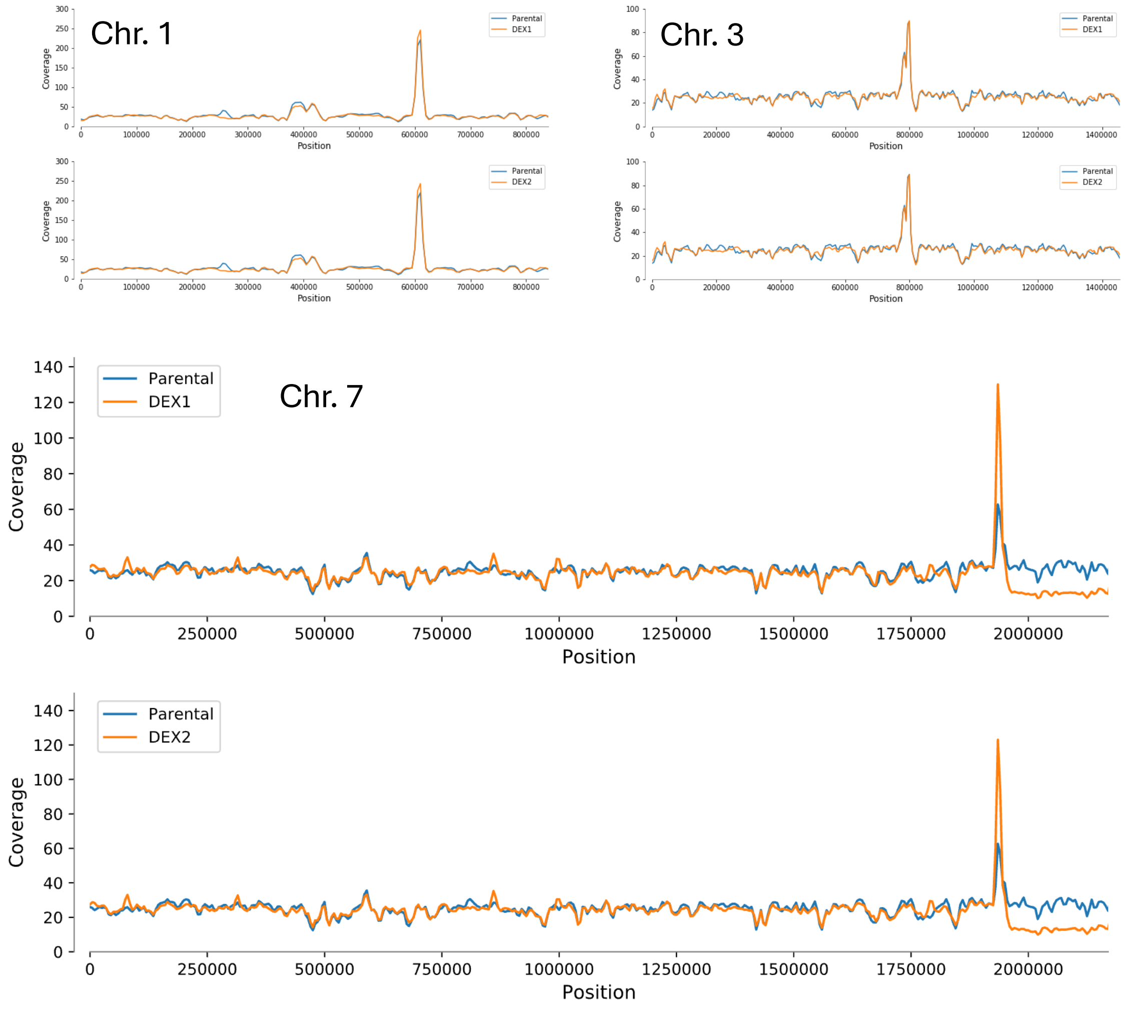

Supplementary Fig. 1: Analysis of DEX cells. (**A**) Immunofluorescence microscopy shows that DEX1 cells express both VSG-2 and VSG-5 at the cell surface. Scale bar, 10 µm. (**B**) *rDNA* promoters, targeted by the *VSG-5* transgene construct are located on chromosomes 1, 3 and 7. The table indicates the fragment size and proportion of each chromosome expected to be lost following transgene integration and *de novo* telomere formation at each site; positions indicated in bp. The lower panels show coverage plots following genome sequencing, revealing partial monosomy on chromosome 7 in both the DEX1 and DEX2 strains, and indicating integration at the site of the *rDNA* promoter on chromosome 7. See Fig. 6A-C.
