# Supplementary material for: A non-coding role for trypanosome VSG transcripts in allelic exclusion
Source: Nucleic Acids Res. 2025 Oct 21;53(19):gkaf1011. doi: 10.1093/nar/gkaf1011 (PMC12539627; doi:10.1093/nar/gkaf1011)
Supplement: gkaf1011_Supplemental_Files [file gkaf1011_supplemental_files.zip › Supplementary Data File 1 description.docx]

**Supplementary Data File 1. RNA-seq and proteomics data and analysis**

Sheet 1: RNAseq analysis: RNAiVSG-2 uninduced v MCPVSG-2 uninduced

Sheet 2: RNAseq analysis: RNAiVSG-2 : 8 h induced v uninduced

Sheet 3: RNAseq analysis: RNAiVSG-2 : 12 h induced v uninduced

Sheet 4: RNAseq analysis: MCPVSG-2 : 8 h induced v uninduced

Sheet 5: RNAseq analysis: MCPVSG-2 : 12 h induced v uninduced

Sheet 6: RNAseq analysis: RNAiVSG-2 8 h induced v MCPVSG-2 8 h induced

Sheet 7: RNAseq analysis: RNAiVSG-2 12 h induced v MCPVSG-2 12 h induced

Sheet 8: Proteomics analysis: RNAiVSG-2 uninduced v MCPVSG-2 uninduced

Sheet 9: Proteomics analysis: RNAiVSG-2: 24 h induced v uninduced

Sheet 10: Proteomics analysis: MCPVSG-2 24 h induced v uninduced

Sheet 11: Proteomics analysis: RNAiVSG-2 24 h induced v MCPVSG-2 24 h induced

Sheet 12: GO term analysis: 200 proteins most significantly reduced in abundance from sheets 9 and 10
